# Technical note: A wearable radiation measurement system for collection of patient‐specific time‐activity data in radiopharmaceutical therapy: system design and Monte Carlo simulation results

**DOI:** 10.1002/mp.15311

**Published:** 2021-11-23

**Authors:** Silvio Morganti, Francesco Collamati, Riccardo Faccini, Giuseppe Iaccarino, Carlo Mancini‐Terracciano, Riccardo Mirabelli, Francesca Nicolanti, Massimiliano Pacilio, Antonella Soriani, Elena Solfaroli‐Camillocci

**Affiliations:** ^1^ National Institute of Nuclear Physics INFN, Rome Italy; ^2^ Department of Physics Sapienza University of Rome Italy; ^3^ Laboratory of Medical Physics and Expert Systems IRCCS Regina Elena National Cancer Institute Rome Italy; ^4^ Medical Physics Division Azienda Ospedaliera‐Universitaria Policlinico Umberto I Rome Italy; ^5^ Specialty School of Medical Physics Sapienza University of Rome Rome Italy

**Keywords:** individualized treatment planning, in vivo dosimetry, molecular radionuclide therapy

## Abstract

**Purpose**: A high level of personalization in Molecular Radiotherapy (MRT) could bring advantages in terms of treatment effectiveness and toxicity reduction. Individual organ‐level dosimetry is crucial to describe the radiopharmaceutical biodistribution expressed by the patient, to estimate absorbed doses to normal organs and target tissue(s). This paper presents a proof‐of‐concept Monte Carlo simulation study of “WIDMApp” (Wearable Individual Dose Monitoring Apparatus), a multi‐channel radiation detector and data processing system for in vivo patient measurement and collection of radiopharmaceutical biokinetic data (i.e., time‐activity data). Potentially, such a system can increase the amount of such data that can be collected while reducing the need to derive it via nuclear medicine imaging.

**Methods**: a male anthropomorphic MIRD phantom was used to simulate photons (i.e., gamma‐rays) propagation in a patient undergoing a ${}^{131}$I thyroid treatment. The administered activity was set to the amount usually administered for the treatment of differentiated carcinoma while its initial distribution in different organs was assigned following the ICRP indications for the ${}^{131}$I biokinetics. Using this information, the simulation computes the Time‐dependent Counts Curves (TCCs) that would have been measured by seven WIDMApp‐like sensors placed and oriented to face each one of five emitting organs plus two thyroid lobes. A deconvolution algorithm was then applied on this simulated data set to reconstruct the Time‐Activity Curve (TAC) of each organ. Deviations of the reconstructed TACs parameters from values used to generate them were studied as a function of the deconvolution algorithm initialization parameters and assuming non‐Poisson fluctuation of the TCCs data points.

**Results**: This study demonstrates that it is possible, at least in the simple simulated scenario, to reconstruct the organ cumulated activity by measuring the time dependence of counts recorded by several detectors placed at selected positions on the patient's body. The ability to perform in vivo sampling more frequently than conventional biokinetic studies increases the number of time points and therefore the accuracy in TAC estimates. In this study, an accuracy on cumulated activity of 5% is obtained even with a 20% error on the TCC data points and a 50% error on the initial guess on the parameters of the deconvolution algorithm.

**Conclusions**: the WIDMApp approach could provide an effective tool to characterize more accurately the radiopharmaceutical biokinetics in MRT patients, reducing the need of resources of nuclear medicine departments, such as technologist and scanner time, to perform individualized biokinetics studies. The relatively simple hardware for the approach proposed would allow its application to large numbers of patients. The results obtained justify development of an actual prototype system to characterize this technique under realistic conditions.

## 1 INTRODUCTION

The accurate determination of absorbed dose in Molecular Radiotherapy (MRT) is essential to increase therapeutic effectiveness, optimize treatment planning, and to evaluate dose‐effect relationships for tumor and normal tissues. Moreover, the European regulation (EU Directive 59/2013) considers mandatory treatment planning and verification in all patients undergoing radiotherapy procedures, including MRT.

Despite several improvements in activity quantification and dosimetric calculations (e.g.^1^), the determination of individual biokinetics remains to date the most challenging issue in MRT. To adequately characterize time‐activity data for radiopharmaceutical dosimetry, the following guidelines^2^ have been proposed:

1. The observation time must be long enough (from 2 to 5 effective half‐time) to characterize the long‐term retention;
2. each exponential component in the model requires at least two data points to be adequately characterized;
3. both the uptake and the washout phases must be identified.

The resulting time‐activity data are then integrated either analytically or numerically to derive the total number of disintegrations in the various source regions of the body.

The foregoing sampling requirements are rarely satisfied, however, in part because of issues related to patient and scanner availability and patient compliance. The second requirement may be critical, mainly due to the lack of a priori knowledge of the number of exponential functions to be used. Several statistical tests for selecting the optimal pharmacokinetics model are available,^3^, ^4^, ^5^ but their use generally requires large numbers of sampled data. Finally, the third requirement also may create some difficulty, especially for rapid uptake.^2^

Due to all these issues, the uncertainty on internal dosimetry calculations for diagnostic or therapeutic studies can still reach 30–100%,^6^ mainly due to uncertainty in biokinetic measurements.

Nowadays, the availability of miniaturized, high‐efficiency photon detectors,interfaced to widely available personal computers, makes possible the design of new systems and new approaches to improve individual dosimetry for routine clinical activities.

WIDMApp (Wearable Individual Dose Monitoring Apparatus) is an attempt to exploit this capability. This system, now at the design stage, aims to provide a precise characterization of the TAC of any organ of interest starting from Time‐Counts Curves (TCCs) acquired with several small, light‐weight radiation detectors worn by the patient during the treatment period. An analytical method, based on a single SPECT/CT scan and an individual Monte Carlo (MC) simulation of radiations propagation in the body, is then used to deconvolve the detected signals and reconstruct the actual drug biokinetics in the patient organs.

The WIDMApp approach is expected to provide a more accurate characterization of the TAC as, in principle, the TCC s sampling frequency and acquisition time span can be set arbitrarily, with frequencies higher than those achievable by serial (i.e., discrete) scanner‐based measurements.

This paper presents and discusses a proof‐of‐concept study of this approach based on an MC simulation. The most relevant sources of uncertainty in characterizing TACs are also discussed.

## 2 MATERIALS AND METHODS

### 2.1 The WIDMApp system

**FIGURE 1:**
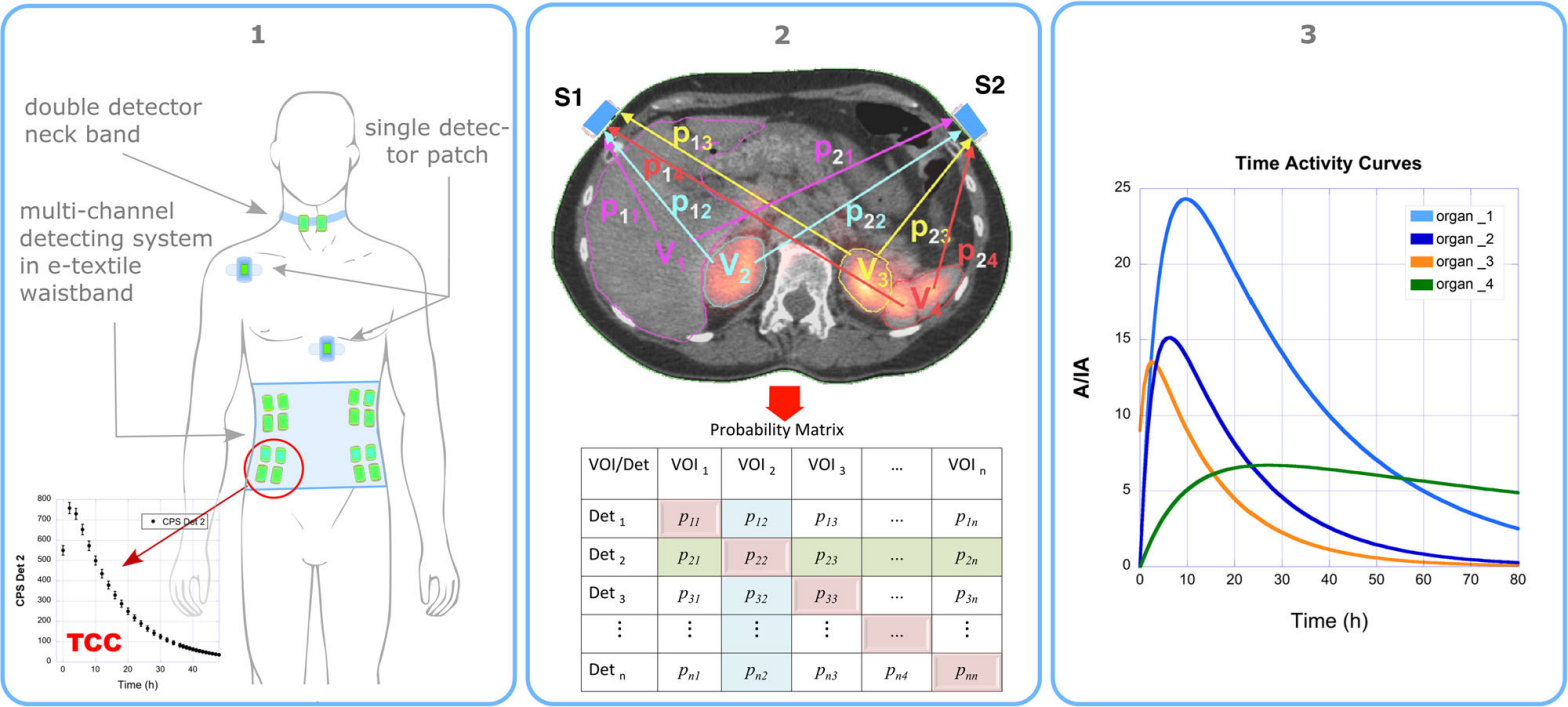
Left: example of detectors grouping and positioning in a wearable radiation‐counting system. Each sensor (or sensors cluster) continuously record the TCC at their site. Center: The probability of each relevant source organ to induce a signal in each sensor/cluster is calculated with an MC simulation using actual patient CT and SPECT. Right: A deconvolution procedure uses the TCCs and the probability matrix to give the best estimate of TACs of any organ of interest

A diagram of the WIDMApp paradigm is presented in Figure 1. It was designed based on the following three considerations:

1. To provide a wearable radiation counting system to continuously record the TCC. The detector elements, or sensors, are stably positioned within compartment of a wearable garment. Each sensor is placed to maximize its sensitivity to an organ of interest, even though it will generally detect radiations emanating from positions throughout the body as shown in Figure 1. The sensors can be used individually or grouped in any number to cover, as a unique subsystem, a wider area. Data are acquired at a fixed or variable sampling frequency during the entire MRT treatment and even continuously over 24 hours per day.
2. To calculate, by means of an MC simulation tailored to each patient, the probability of each source organ to induce a signal in each target sensor.
  The MC uses Computed Tomography (CT) data to simulate the patient anatomy and the Single Photon Emission Computed Tomography (SPECT) image to reproduce the activity distribution within individual organs and the whole body.
3. To provide a data deconvolution procedure that uses the measured TCCs and the probability coefficients calculated with the MC simulation to give the best estimate of TACs of any organ of interest.

### 2.2 Feasibility study

The innovative aspect of the WIDMApp concept is the ability of being able to reconstruct an organ's TAC starting from a relatively small number of sensors whose signal is determined by the local superimposition of radiations generated (almost) anywhere in the patient's body.

The feasibility of this approach has been studied on a simplified model of a ${}^{131}$I thyroid treatment. An MC simulation of a male anthropomorphic phantom has been used to simulate the propagation of particles originating in five source organs (liver, kidneys, spleen, and bladder) plus the thyroid, and to evaluate the probability they were detected by seven WIDMApp‐like sensors: two sensors, one per lobe, placed on the thyroid, the remaining five positioned and oriented toward the center of each of the foregoing organs. Realistic values for the initial activities ($A_{0}$) and the effective half‐lives ($t_{1/2}^{\text{eff}}$) have been assigned to each organ to generate the TCC s that would have been measured by each sensor continuously over 10 days. A mono‐exponential function decay model, sampled at least once per hour, has been assumed for every organ.

The WIDMApp data analysis tool has been then applied to this simulated data set to perform the deconvolution of the TCCs and to reconstruct the individual TAC of each organ. Deviations of the reconstructed $A_{0}$ and $t_{1/2}^{\text{eff}}$ TAC parameters from their actual values have been studied, organ by organ, varying the initial guess on the TACs parameters used to initialize the data analysis algorithm and assuming non‐Poisson fluctuations of the simulated TCC s data points.

#### 2.2.1 Monte Carlo simulation

The MC simulation has been developed using Geant4,^7^, ^8^ one of the most commonly used tool‐kits to simulate the interaction of radiation with matter. Electromagnetic interaction, in particular, has been simulated with *emstandard_opt4*, the most accurate low energy model package among the standard electromagnetic physics lists available in the Geant4 package. All these models are regularly benchmarked for medical applications.^9^

**FIGURE 2:**
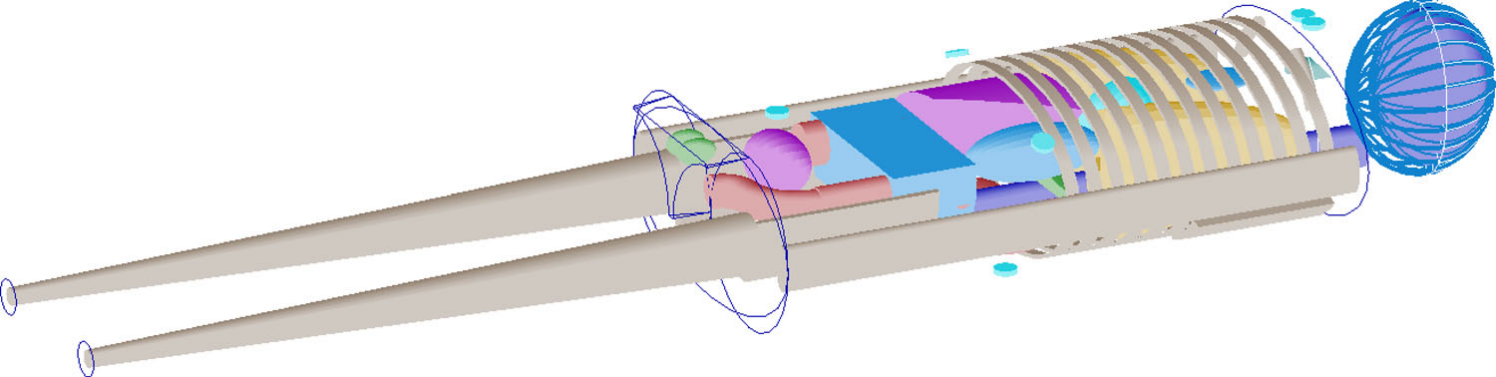
MIRD male anthropomorphic phantom used for the MC simulation. The soft tissue around the organs is not shown to make internal organs visible; the outer soft tissue profile is indicated by the blue lines visible at the neckline and the legs attachment. The seven small cyan cylinders nearby the phantom represent the WIDMApp detectors included in the simulation

The patient anatomy has been described using the simple representation of a human body, mimicking an adult man weighing 70 Kg, provided by the MIRD (Medical Internal Radiation committee) phantom^10^ shown in Figure 2.

This stylized anthropomorphic phantom includes multiple internal organs, each represented as a single, regularly shaped volume. Each organ is considered homogeneous both in composition and density and is made up of one out of three types of tissue: lung, bone, and generic soft tissue. The human body shows the arms parallel to the torso and the legs extended, simulating the typical positioning of a patient undergoing a CT scan. More details about the Geant4 implementation of the MIRD anthropomorphic phantom can be found in Ref. [11]

Seven WIDMApp detectors, also shown in Figure 2, have been included in the simulation as simple hollow cylinders, 3 mm in height by 13 mm in radius. For the purpose of this study, they are used only to evaluate the passage of radiation through the detector element in that particular position. For each detector, the counting rate has been then defined as the number of particles entering its volume (counts) in one second assuming therefore a 100% detection efficiency (i.e., the counts equal the number of particle crossing the detector independently of their path or energy). It should be noted that the latter quantity is simply a counts scale factor that does not bias the measurement of the time evolution of the TCCs. Depending on the isotope used and/or each time the detectors had different characteristics, the actual detection efficiency, for each sensor, can be adequately taken into account by applying a scale factor to each of the measured TCCs.

All organs have been considered to be uniformly active, that is, the position where the primary particle starts has been sampled uniformly within the whole organ volume.

To speed up the simulation, only the 364‐keV photon produced in the ${}^{131}$Xe${}^{★}$ de‐excitation, which is, in turn, the final state of the $\beta^{-}$ decay of ${}^{131}$I, have been generated. Both the ${}^{131}$I $\beta^{-}$ decay with an end‐point energy of 606 keV and Bremsstrahlung emission have been neglected. The latter has been considered negligible, while most of the electrons in that energy range are absorbed by the tissue and cannot reach the detector.

Six different simulations, each simulating particles emitted by a single source organ, have been performed. Every simulation gives the probability that a primary particle generated within the activated organ produces, directly or indirectly, a signal in each detector.

The result is a 6×7 matrix (six organs, seven detectors) whose elements $p_{ij}$ (see the central panel of Figure 1), indicate the probability that a decay in the jth organ yields a signal in the ith detector.

It has to be noted that the probability matrix elements are set only by the patient anatomy, the emitted particle type, and their energy spectrum. The $p_{ij}$ values thus do not depend on the biokinetics of the radiopharmaceutical or the administered activity.

#### 2.2.2 TCC generation

Assuming the pharmaceutical uptake of each organ as an instantaneous accumulation followed by a mono‐exponential decay function, the time‐dependent signal, $\left(S_{i}\left(t\right)\right)$, generated by each detector is

(1) $$S_{i}\left(t\right)=\sum_{j}p_{ij}\;A_{0j}\exp\left(-\frac{t}{t_{{1/2}_{j}}^{\text{eff}}/\ln\left(2\right)}\right),$$

where $p_{ij}$ are the probability matrix elements, $A_{0j}$ the activity of the j organ at t=0, and $t_{{1/2}_{j}}^{\text{eff}}$ the effective half‐life of the radiopharmaceutical in that organ.

To assign realistic values to the $A_{0j}$, the administered activity has been set to 2.5 GBq, equal to the amount usually administered for the treatment of differentiated carcinoma. Following the general indications of the ${}^{131}$I biokinetics model from ICRP,^12^ roughly 30% of this amount has been assigned to the thyroid and the 60% has been ascribed to the source organs proportionally to their mass and assuming the same activity concentration. The remaining 10% has been considered to be uniformly distributed in the remainder of the body and eliminated almost instantly.

The effective half‐life $t_{{1/2}_{j}}^{\text{eff}}$ values have been set combining the ${}^{131}$I physical half‐life with the assumption on the biological half‐lives $t_{{1/2}_{j}}^{\text{bio}}$ shown in Table 1.

**TABLE 1:** Initial activities $A_{0}$ and biological, $t_{1/2}^{\text{bio}}$, and effective, $t_{1/2}^{\text{eff}}$, decay times assigned to each organ

Organ	$A_{0}$ [MBq]	$t_{1/2}^{\text{bio}}$ [h]	$t_{1/2}^{\text{eff}}$ [h]
Thyroid	750.0	1920.0	174.9
Right Kidney	115.0	12.0	11.3
Left Kidney	105.0	10.0	9.5
Bladder	35.0	7.0	6.8
Liver	1115.0	24.0	21.3
Spleen	130.0	17.0	15.6

The experimental TCCs have been then generated from Equation (1) with the parameters shown in Table 1.

Data have been generated to simulate a measurement period lasting 240 hours (10 days after administration). Exploiting the flexibility of the WIDMApp system, the acquisition has been split into two phases with different sampling rates; one measurement every 12 minutes for the first 48 hours and one measurement per hour for the next 8 days. In this way, both the fast wash out of the source organs and the slower thyroid clearance $t_{1/2}^{\text{eff}}$ can be characterized with about the same number of data points. Each measurement corresponds to the counts acquired by the sensor in one second.

To reproduce the Poisson fluctuations of real measurements, any value calculated with Equation (1) have been dispersed according to a Poisson distribution with mean and variance set by its value at the time t.

**FIGURE 3:**
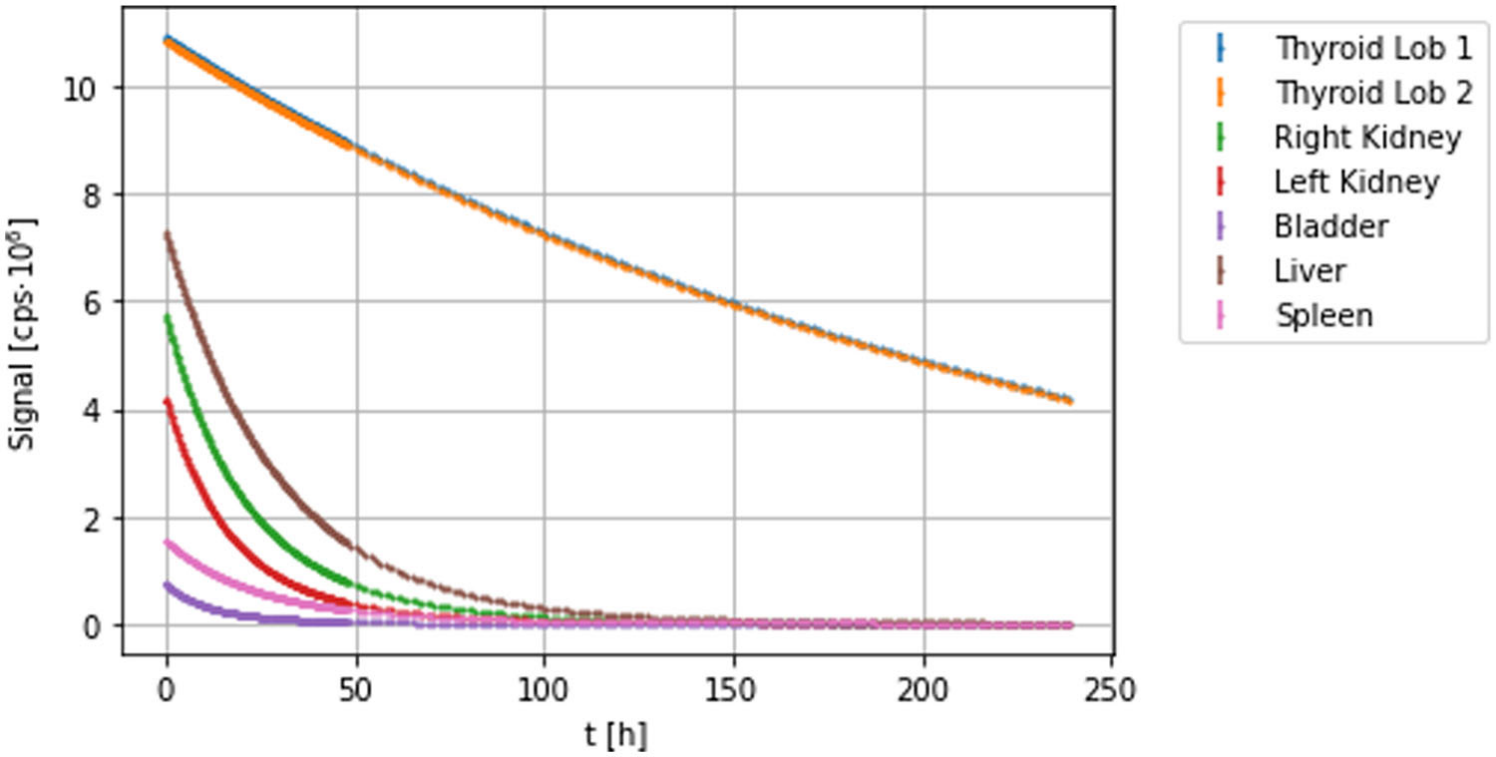
The seven simulated TCCs

The seven TCCs, one per detector, are shown in Figure 3.

### 2.3 The deconvolution algorithm

**FIGURE 4:**
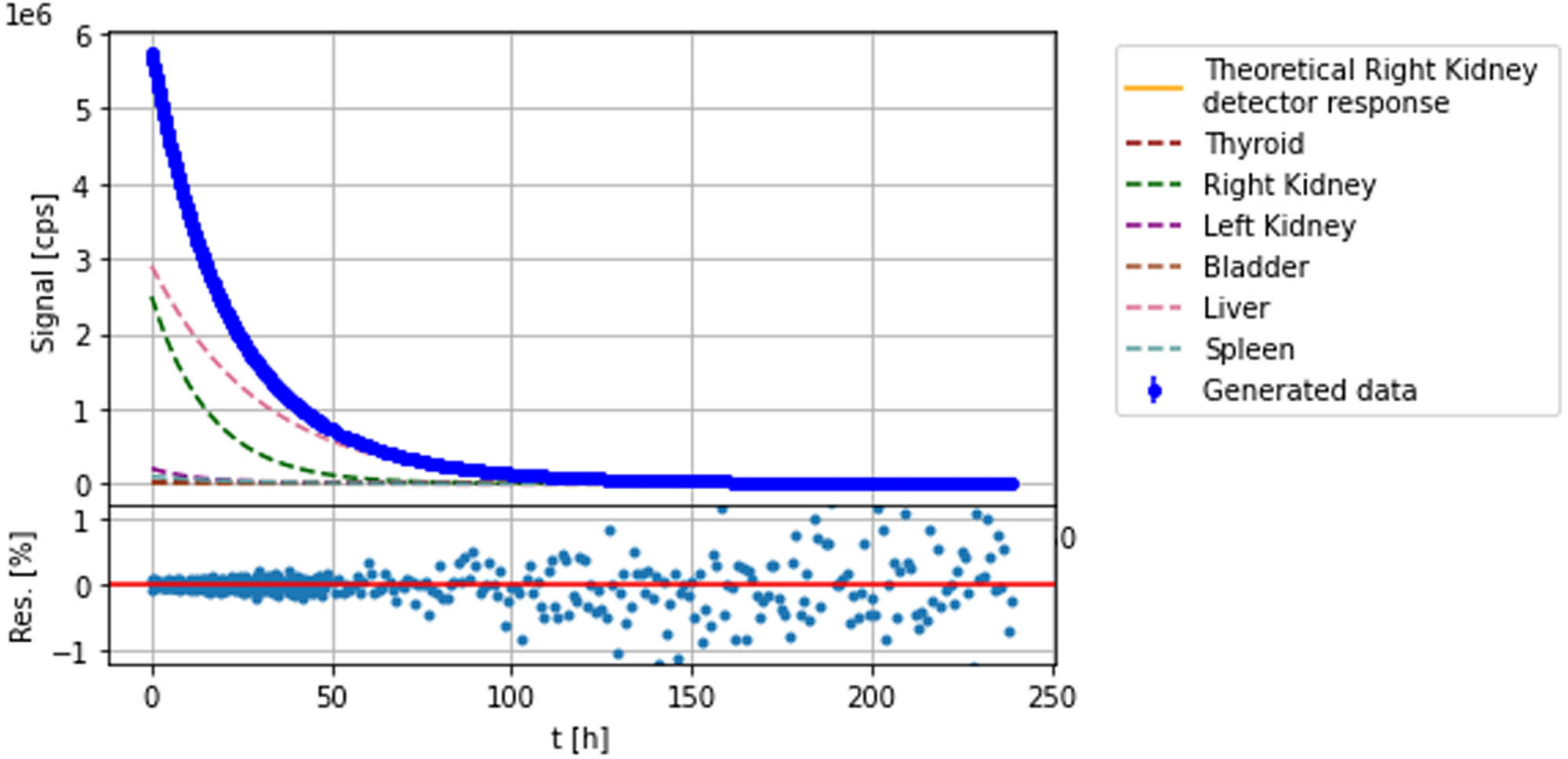
One of the TCC output of the Monte Carlo simulation. The blue dots are the simulated signal recorded by the detector facing the right kidney. The colored curves show the contribution of each of the six organs to this signal. The bottom panel shows the residuals between each point and the exponential function result of the Equation (1). It highlights the fluctuations added to simulate the Poisson dispersing

Each of the seven generated TCCs is the result of the superimposition of the signals sourced by all the six organs as shown in Figure 4. To reconstruct the organ individual TAC, a deconvolution algorithm has been used to identify the combination of six pairs of parameters, one for each organ, $A_{0j}^{\prime }$ and $t_{{1/2}_{j}}^{\prime \text{eff}}$, that best reproduces all the seven TCCs data points at the same time. Given the simple biokinetics modeling this study is based on, this is achieved by defining a merit function $\chi^{2}\left(A_{0j}^{\prime },{t_{1/2}^{\prime }}_{j}\right)$ that considers all points from all the seven TCCs:

(2) $$\begin{array}{c} \chi^{2}\left(A_{0j}^{\prime },{t_{1/2}^{\prime }}_{j}\right)=\sum_{i}^{N_{\text{det}}}\sum_{k}{\left(\frac{S_{i}\left(t_{k}\right)-S_{i}\left(t_{k};A_{0j}^{\prime },{t_{1/2}^{\prime }}_{j}\right)}{\sigma_{i}\left(t_{k}\right)}\right)}^{2}, \end{array}$$

where $S_{i}\left(t_{k}\right)$ is the experimental TCC value of detector i at the time $t_{k}$ (Equation (1)) and $S_{i}\left(t_{k};A_{0j}^{\prime },{t_{1/2}^{\prime }}_{j}\right)$ is the test value the signal will be compared with. The same probability matrix elements used to generate the TCCs have been used to define the merit function. Finally, $\sigma_{i}\left(t_{k}\right)$ is the standard deviation estimate for each measurement.

It has to be noted that the free parameters of Equation (2) are strongly intercorrelated. Therefore, to perform this nontrivial minimization of $\chi^{2}\left(A_{0j}^{\prime },t_{{1/2}_{j}}^{\prime }\right)$, several algorithms available in the SciPy library^13^ plus Minuit2^14^ have been tested finding the Nelder–Mead algorithm^15^ as the most stable.

### 2.4 Stability of the deconvolution algorithm

The accuracy of the Nelder–Mead algorithm in reconstructing the true $A_{0_{j}}$ and $t_{{1/2}_{j}}^{\text{eff}}$ has been studied as a function of both:

- the initial values $A_{0_{j}}^{\prime }$ and $t_{{1/2}_{j}}^{\prime \text{eff}}$ used as starting point of the deconvolution algorithm, to which we will refer as *priors* in the following;
- the presence of random fluctuation, larger than that associated with the standard Poisson distribution, on the actual value of the TCC experimental data.

More in detail: the effect of an uncertainty on the priors has been estimated repeating the deconvolution algorithm 100 times sampling each time the priors $S_{i}\left(t_{k};A_{0_{j}}^{\prime },{t_{1/2}^{\prime }}_{j}\right)$ from a uniform distribution centered on their true value. The process has been repeated nine times varying the half‐width of the distribution from 10% to 90% of the true value in steps of 10%. For each iteration, the results have been expressed in terms of the mean value and width of the distribution of the residuals, $\left(A_{0_{j}}^{\prime }-A_{0_{j}}\right)$ and $\left({t^{\prime }}_{{1/2}_{j}}^{\text{eff}}-{t_{1/2}^{\text{eff}}}_{j}\right)$.

The same quantities have been used to quantify the effect of non‐Poisson fluctuation of the simulated data. To this aim, the deconvolution algorithm has been applied on eight different data sets. Each data set has been obtained dispersing the data points by adding a quantity sampled from a uniform distribution centered on zero and having half‐width equal to a fixed fraction of the data point central value. The minimization procedure has been repeated 100 times per data set, with half‐width ranging from 5% to 40% in steps of 5% (i.e., nine times). In this case, all the 100 minimization procedures started with the same priors, obtained sampling their values only one time from a uniform distribution centered on the MC assigned value and with a half‐width of 50%.

## 3 RESULTS

The probability matrix calculated by the MC on the MIRD phantom anatomy is shown in Table 2. By rows, it shows the probability that particles sourced by any of the six organs i reach one of the detector j. By column, it shows the probability with which each organ contributes to the signal of the seven detectors.

**TABLE 2:** Probabilities of each source organ (columns) to produce a signal in each target detector (rows). The color scale provides a visual feedback of the probabilities reported numerically in the cells. Considering the two detectors faced to the thyroid as a single system, then the matrix is a square matrix and the terms on the diagonal are the probability that the radiation produced by each organ is detected by the closest detector (or system of detectors, for the thyroid) The uncertainties have been calculated by performing 100 simulations for each configuration. This matrix is referred in the text and equations as matrix $p_{ij}$

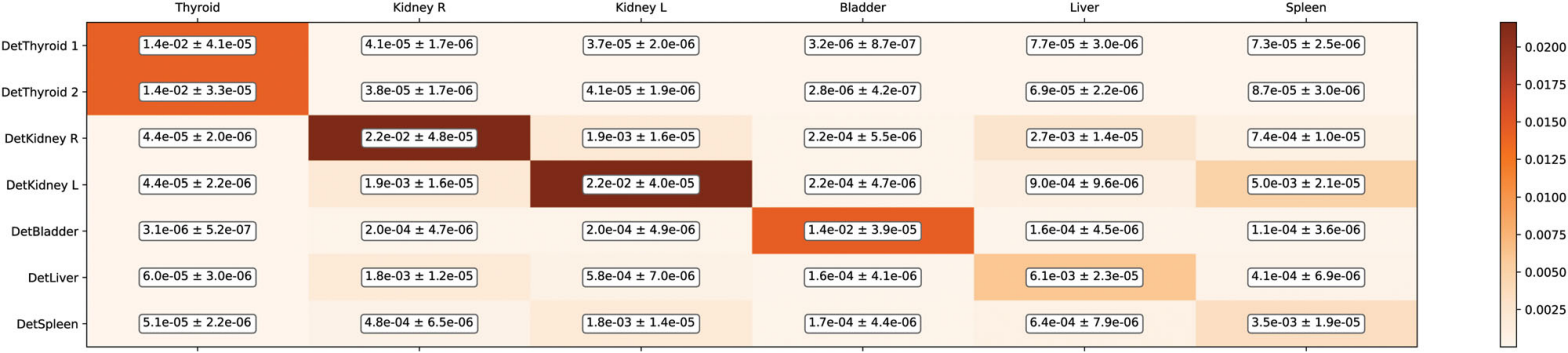

The color scale highlights that the matrix is substantially diagonal, that is, most of diagonal elements are two order of magnitude greater than nondiagonal ones: this indicates that each detector is most sensitive to the signal coming from the organ above which it has been placed. Liver and Spleen detectors are two notable exception, showing almost the same sensitivity to the organs on which they are positioned and the kidneys behind them.

### 3.1 TAC reconstruction

The convergence to a minimum of the merit function (Equation 2) has been tested applying the Nelder–Mead algorithm. As for all the minimization procedures, the convergence to a stable minimum and the errors on the corresponding function parameters strongly depends on the priors the minimization process starts with.

#### 3.1.1 Stability of TAC parameters with respect of priors

**FIGURE 5:**
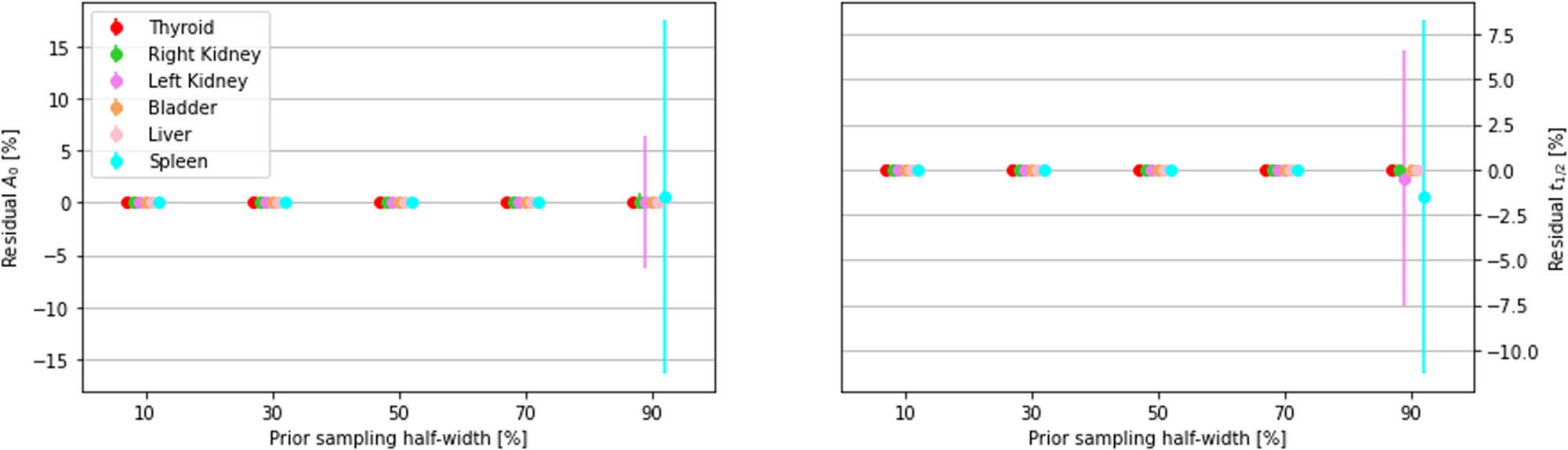
Differences, expressed as a percentage, between the values $A_{0}$ (left panel), $t_{{1/2}_{j}}^{\text{eff}}$ (right panel) set in Table 1 and $A_{0}^{\prime }$, ${t_{1/2}^{\prime }}_{j}^{\text{eff}}$ (output by the minimization procedure) as a function of the width of the uniform distributions from which the priors are sampled. The residuals are shown for nine values of the sampling distribution half‐width starting from a minimum of 10%. For each sampling width a group of six dots shows the mean value and dispersion of the six organs considered in the MIRD simulation

The initial choice of $A_{0_{j}}^{\prime }$ and $t_{{1/2}_{j}}^{\prime \text{eff}}$ can be quite far from the true values (in this study those reported in Table 1). The robustness of the minimization procedure, in terms of the difference between the priors used to start the process and the parameters the minimization algorithm converge to, are summarized in Figure 5.

The minimization algorithm converges always to the same true values (Table 1), even starting with priors up to 80% higher/lower than the truth. For larger differences, however, the minimization does not always manage to find the correct parameters. In particular, large fluctuations are observed in the $t_{{1/2}_{j}}^{\prime \text{eff}}$ and $A_{0}^{\prime }$ values of the spleen TAC and in the left kidney and bladder washout rate. In these cases, a systematic overestimate of the $A_{0}^{\prime }$ and underestimate of the $t_{{1/2}_{j}}^{\prime \text{eff}}$ are observed.

#### 3.1.2 Stability of the TAC parameters with respect to data dispersing

Many effects may introduce random fluctuations, larger than those based on the standard Poisson distribution, on the actual value of the TCC experimental data with a potential effect on the errors on reconstructed TAC parameters. Any movement causing small variation of the sensor field of view, like patient breathing, walking, or drift in the electronics response due to environmental temperature changes could cause such an effect. To model a worst‐case scenario, fluctuations following a uniform distribution have been applied to each simulated datum.

**FIGURE 6:**
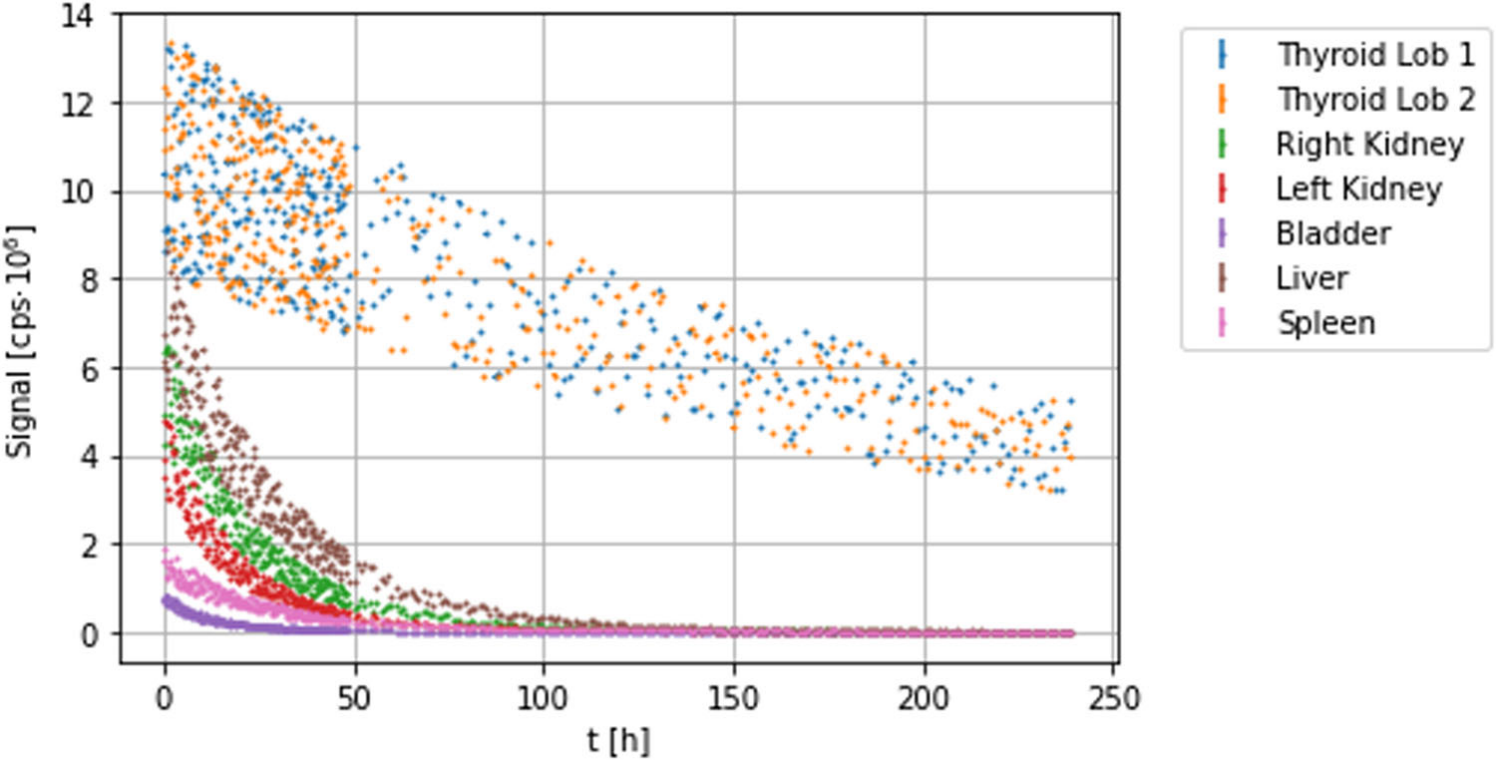
Twenty‐five percent dispersed TCCs. To each data point ($S_{i}\left(t\right)$) a random number sampled from a uniform distribution with half width 25% of its original value has been summed to simulate non‐Poisson fluctuation on the experimental data. The different data points density after the first 50 hours is due to the different sampling frequency the data have been generated with, as explained in the text

Figure 6 shows, as an example, the seven TCCs obtained once the original data points have been dispersed adding a random number sampled from an uniform distribution with half width of 25% of their central values.

**FIGURE 7:**
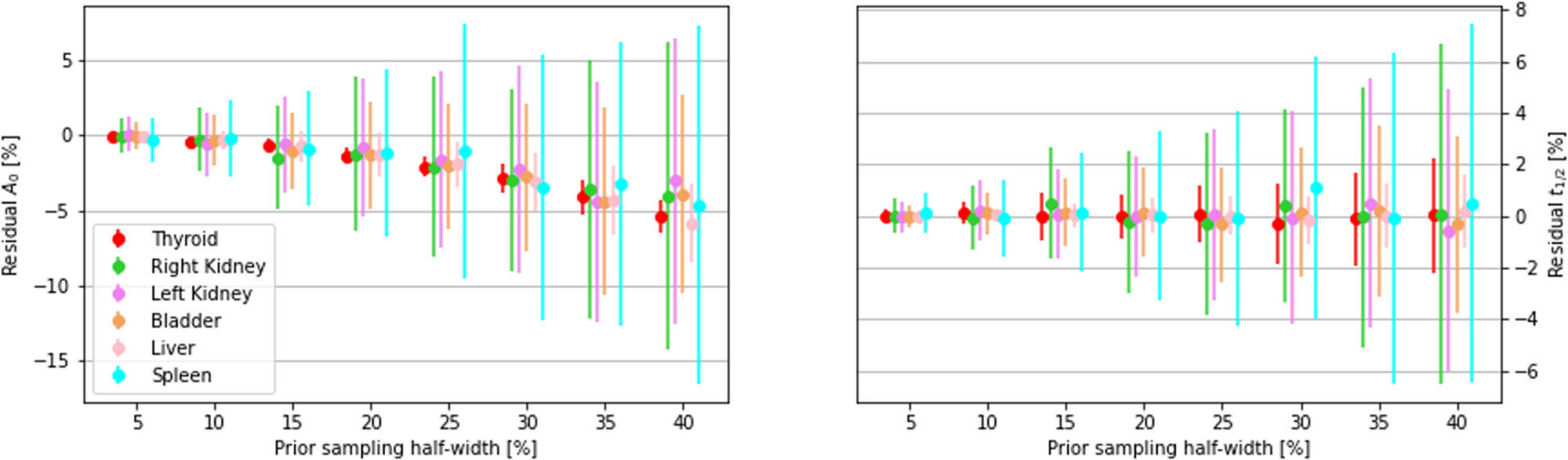
Differences, expressed as a percentage, between calculated and true values of the initial activities (left panel) and the half‐lives (right panel) for the eight data dispersions considered. For each of the eight assignments of the uniform sampling distribution half‐width, a group of six dots shows the mean value and dispersion of the residuals on each of the six organs considered in the MC simulation. Priors have been set, once for all, sampling a 50% half‐width uniform distribution

Results are shown in Figure 7 as the percentage difference on the fit parameters $A_{0j}$ and $t_{{1/2}_{j}}^{\text{eff}}$ in the eight noisy simulated scenarios with respect to the biokinetics assignment of the adopted modeling (Table 1). The minimization algorithm manages to converge to the true TAC parameters within 1% error up to an uniform dispersion of the data points of half‐width ≤ 20 %. Higher fluctuations do not change much the shape of the TAC s: $t_{{1/2}_{j}}^{\text{eff}}$ residuals are always smaller than 7%. The $A_{0j}$ values instead are systematically underestimated, with an increasing trend as the extent of the distribution increases, introducing an offset in the determination of the curve integral.

#### 3.1.3 Stability of organ cumulated activity estimate

**FIGURE 8:**
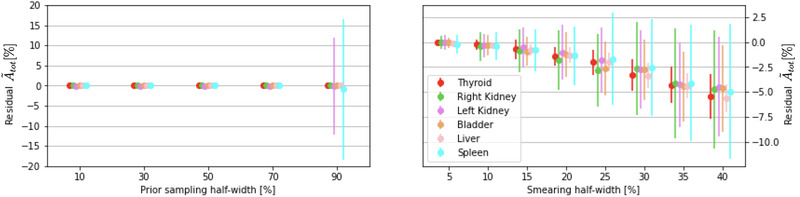
Residuals on the organ cumulated activities ${\tilde{A}}_{\text{tot}}\left[\%\right]$ calculated as the percentage variation between the experimental TCC integrals and their true values

The uncertainty on the TAC parameters, and therefore the organs' cumulated activities (total number of disintegration occurred in a target organ from time = 0 to time = ∞), depends on the combined effects of the priors choice and the data fluctuations. The effect of these two sources of uncertainties on the cumulated activity is shown in Figure 8 as the residuals in percentage, ${\tilde{A}}_{\text{tot}}\%$, between the calculated and actual cumulated activity.

These plots confirm that the data fluctuations dominate the accuracy with which the cumulated activity is measured.

Nevertheless, given the range of priors and data dispersion considered in this study, the overall accuracy on the TAC integral is of the order of ±5% with data dispersing (non‐Poisson random fluctuation averaging to zero and spanning the entire data‐acquisition timeframe) up to ±20%.

## 4 DISCUSSION

The approach described in the current paper, illustrated for Iodine‐131, can be applied to gamma‐ray‐emitting radiopharmaceuticals generally.

A simplification introduced in this study is the basic geometrical description of the patient body provided by the MIRD phantom and the simplest, one detector per organ, configuration of the WIDMApp system. In this case, the main advantage resides in the much reduced computation time needed for the simulation, which comes obviously at the cost of a less accurate reproduction of the problem. As a result, the MC simulation described in this paper required a computation time of the order of 10 minutes on an average commercial‐grade laptop.

The foregoing simplification, however, does not diminish the significance of this study because, in principle, more complex radiopharmaceutical distributions and patient anatomies can be addressed by increasing the number of detectors used. The proposed balance between these two features is adequate to provide the ground‐level proof of principle of the WIDMApp approach and to identify and test the tools that best meet its requirements.

## 5 CONCLUSIONS

“WIDMApp' is a multi‐channel radiation (i.e., photon) detector and data processing system for in vivo patient measurement and collection of radiopharmaceutical biokinetic data (i.e., time‐activity data). Potentially, such a system may increase the amount of such data that can be collected while reducing the need for nuclear medicine imaging to assess the individual biokinetics. Using a limited number of judiciously positioned detectors on the patient's body, as determined by the known biodistribution of the particular radiopharmaceutical under consideration, patient‐specific time‐activity curves and cumulated activities in key source organs can be derived to an accuracy of ±5%, as determined by the Monte Carlo simulations presented. Despite the relative simplicity of the radiopharmaceutical (i.e., ${}^{131}$I‐iodide) kinetics and patient anatomy modeled in the current study, the method described appears fairly robust and flexible and thus merits further development.

## CONFLICT OF INTEREST

The authors have no conflicts to disclose. Data are available on request from the authors.
